# Free flap reconstruction of a cast-related pressure ulcer in a pediatric patient with spinal muscular atrophy

**DOI:** 10.1016/j.jpra.2026.02.007

**Published:** 2026-02-08

**Authors:** İbrahim Faruk Adıgüzel, Mustafa Kemal Yenidünya

**Affiliations:** a Department of Hand Surgery, Etlik City Hospital, University of Health Sciences, Ankara, Türkiye; b Department of Orthopaedics and Traumatology, Etlik City Hospital, University of Health Sciences, Ankara, Türkiye

**Keywords:** Spinal muscular atrophy, Pressure ulcer, Free flap reconstruction, Anterolateral thigh flap

## Abstract

Spinal muscular atrophy (SMA) is a hereditary neuromuscular disorder characterized by progressive lower motor neuron loss, resulting in severe muscle atrophy, fatty degeneration, and impaired tissue perfusion. These changes increase susceptibility to pressure-related soft tissue injuries and delay wound healing.

We report a rare case of free flap reconstruction in a pediatric patient with SMA type 2 who developed a cast-related pressure ulcer. A 9-year-old girl underwent bilateral hamstring release and distal femoral extension osteotomy followed by long leg circular casting. After cast removal, a progressive anterolateral crural pressure ulcer with exposed necrotic muscle developed despite conservative treatment.

Reconstruction was performed using an anterolateral thigh (ALT) free flap. Due to extensive muscle atrophy, fibrosis, and loss of normal anatomical planes, a modified en bloc myocutaneous flap elevation based on antegrade pedicle dissection was used instead of standard perforator-based dissection. The flap was successfully anastomosed to the posterior tibial vessels without complications.

At 6-month follow-up, complete and stable wound healing was achieved. This case emphasizes the reconstructive challenges in SMA patients and demonstrates that free flap reconstruction can be safely performed using adapted surgical techniques.

## Introduction

Spinal muscular atrophy (SMA) is an autosomal recessive neuromuscular disorder caused by mutations in the *SMN1* gene and characterized by progressive lower motor neuron loss.^1^ Patients with SMA type 2 are able to sit independently but are unable to ambulate, and progressive muscle weakness leads to orthopedic deformities, contractures, and an increasing need for surgical interventions.^2^^,^^3^

In SMA, severe muscle atrophy, fatty infiltration, and fibrosis impair local tissue perfusion and reduce the protective cushioning effect of soft tissues, predisposing patients to pressure-related skin and soft tissue injuries and delayed wound healing.^4^ While conservative measures are the cornerstone of management, surgical reconstruction may be required in cases of extensive tissue loss.

SMA-related structural alterations distort normal fascial planes and intermuscular anatomy, complicating local or regional flap planning and occasionally necessitating free flap reconstruction. This report describes a pediatric patient with SMA type 2 who developed a cast-related pressure ulcer requiring free flap reconstruction and highlights disease-specific reconstructive challenges.

## Case presentation

**Figure 1:**
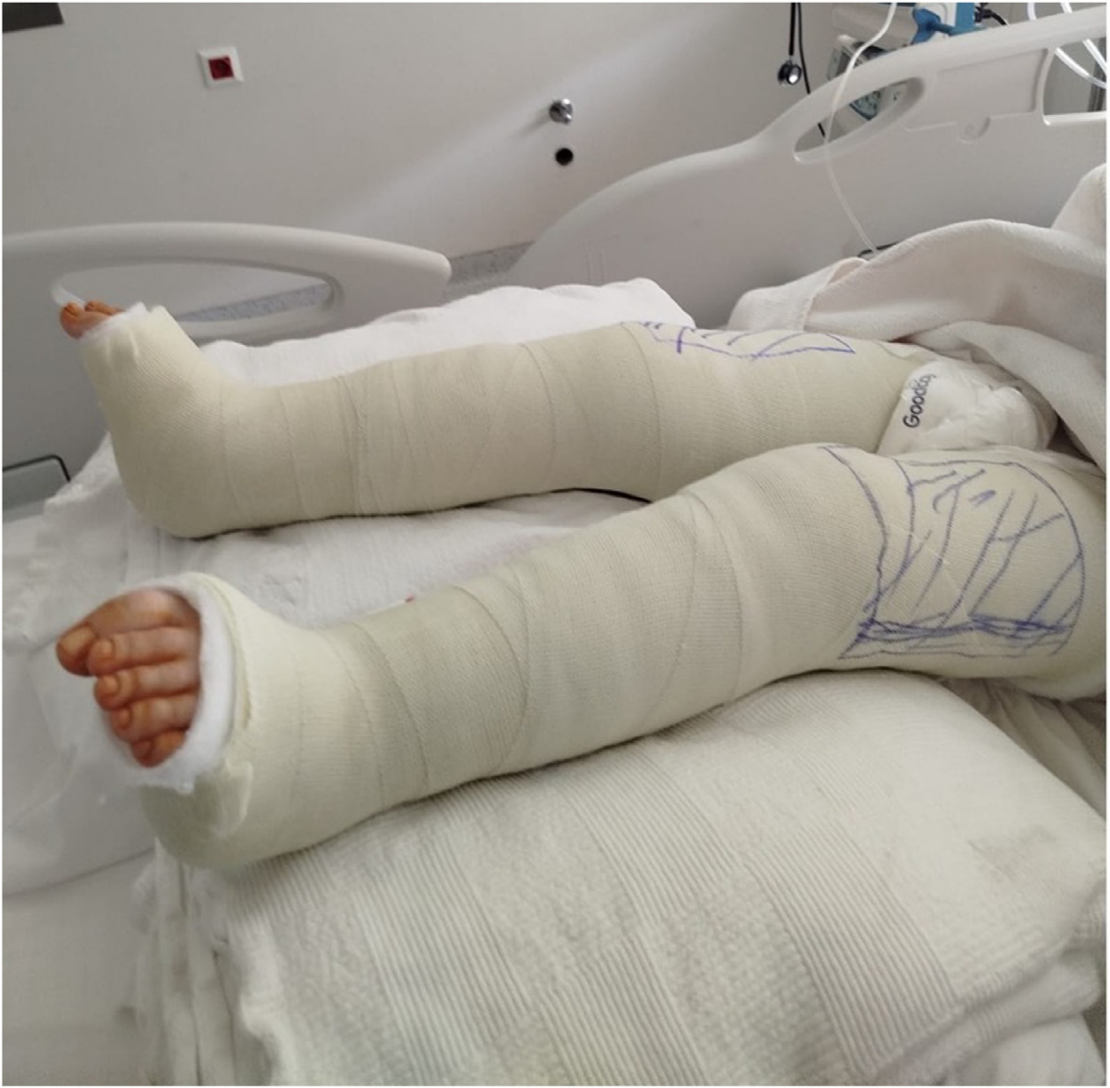
Bilateral long-leg circular cast immobilization after hamstring release and distal femoral extension osteotomy.

**Figure 2:**
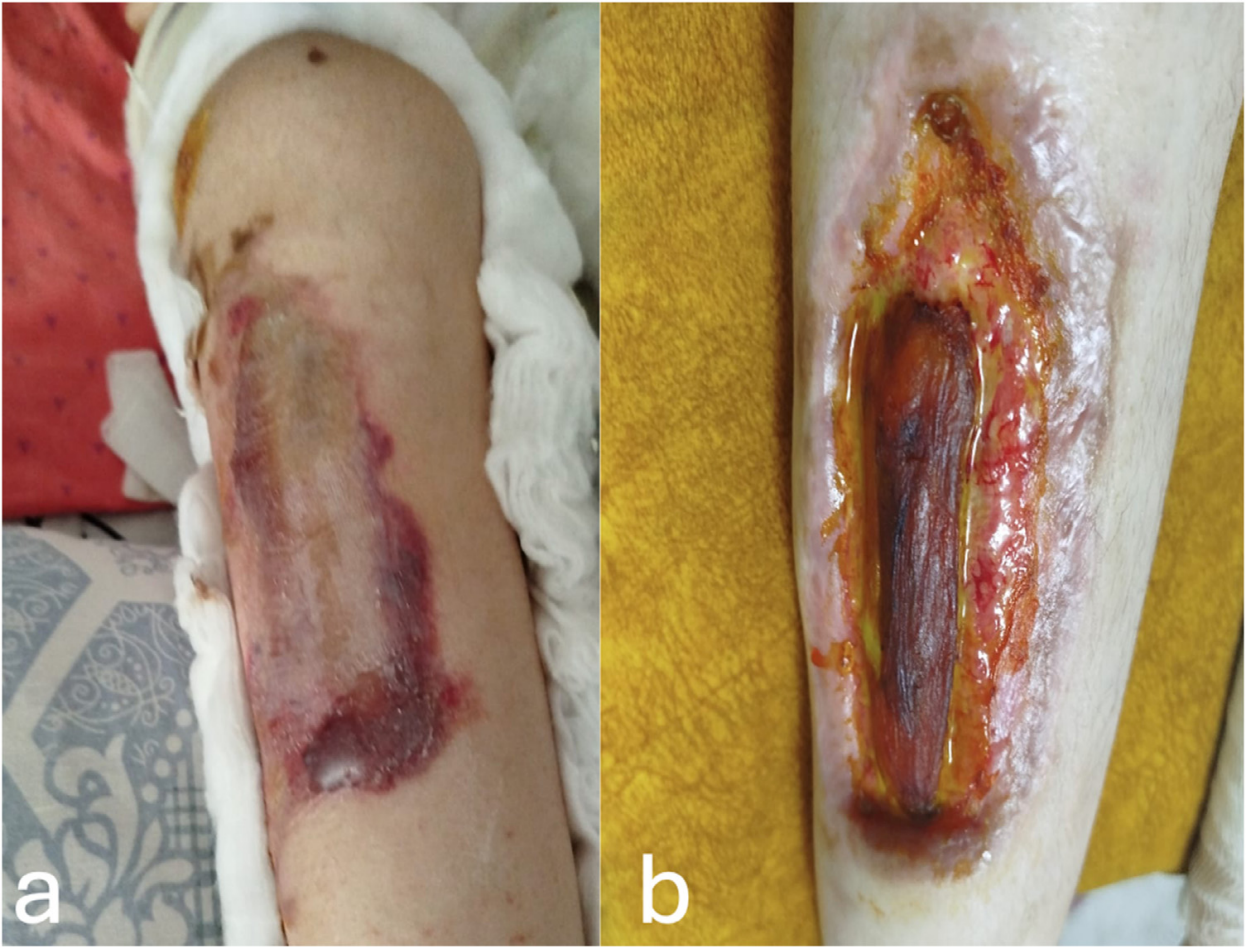
Cast-related pressure ulcer of the anterolateral right cruris: (a) immediately after cast removal; (b) progressive deepening with underlying muscle necrosis during follow-up.

A 9-year-old female patient diagnosed with SMA type 2 underwent bilateral hamstring release and distal femoral extension osteotomy for knee flexion contracture, followed by long leg circular casting (Figure 1). After cast removal, pressure sores were observed, primarily on the crural region Despite 1 month of serial wound care, the anterolateral ulcer on the right leg progressed to a deep soft tissue defect with exposed necrotic muscle, necessitating surgical reconstruction (Figure 2).

Following thorough debridement, the recipient site was carefully assessed with particular attention to vascular accessibility. Severe muscle atrophy, diffuse fatty infiltration, and advanced fibrosis had resulted in a complete loss of normal intramuscular anatomical planes, rendering any form of intramuscular vascular dissection unsafe and technically unfeasible. For this reason, preparation of the anterior tibial artery–vein bundle was abandoned, as neither antegrade nor retrograde-flow anastomotic strategies could be safely pursued without extensive intramuscular dissection in the severely atrophic and fibrotic muscle tissue. Instead, the posterior tibial vascular package was selected as the recipient site, as it allowed reliable vascular control without the need for intramuscular dissection, a critical consideration in the setting of SMA-related muscle pathology.

**Figure 3:**
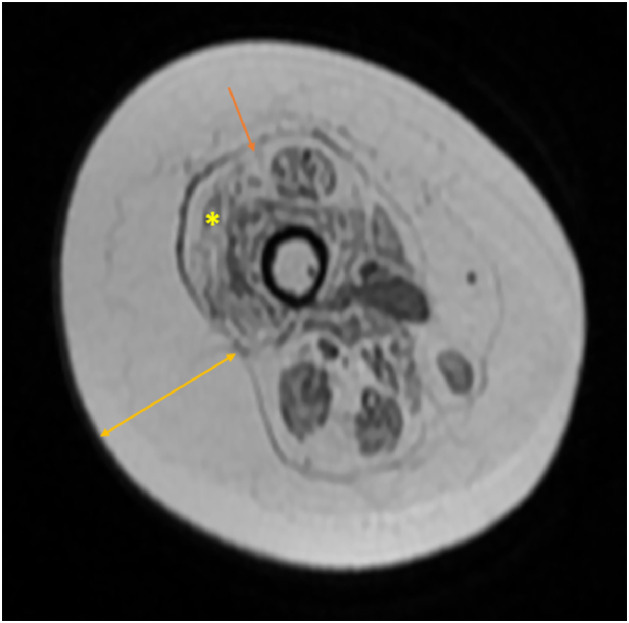
Axial T1-weighted MRI of the mid-thigh demonstrating disease-specific muscular alterations in SMA, including marked atrophy and fatty degeneration of the vastus lateralis muscle (*), blurring of the intermuscular septum (arrow), and increased subcutaneous fat thickness relative to reduced muscle bulk (double-headed arrow).

Similar disease-specific constraints were encountered at the donor site. Given that the posterior tibial vessels of the affected limb were selected as recipient vessels, the anterolateral thigh flap was harvested from the contralateral thigh to allow optimal pedicle orientation and tension-free microvascular anastomosis. Although a dominant perforator was identified using a handheld Doppler device, retrograde intramuscular perforator dissection was not feasible due to severe muscle degeneration and an obscured perforator course. Although not obtained for preoperative planning, axial magnetic resonance imaging of the thigh demonstrated severe muscle atrophy and diffuse fatty infiltration with blurring of normal muscle architecture, reflecting chronic neuromuscular involvement typical of spinal muscular atrophy (Figure 3). Consistent with these imaging findings, extensive muscle atrophy, fibrosis, and loss of normal anatomical planes rendered safe intramuscular perforator dissection unfeasible, as required in the conventional anterolateral thigh flap technique.

Given the impossibility of retrograde perforator tracking, the reconstructive strategy was necessarily modified to a pedicle-oriented approach. The incision was extended proximally to expose the descending branch of the lateral femoral circumflex artery beneath the rectus femoris muscle. From the bifurcation of the lateral femoral circumflex artery, antegrade dissection of the descending branch was performed, and the flap was elevated en bloc together with a limited segment of the vastus lateralis muscle, incorporating the vascular pedicle within the muscle tissue. This approach avoided hazardous intramuscular perforator skeletonization and ensured pedicle reliability in the presence of advanced SMA-related muscle pathology. Microvascular anastomosis was performed using an end-to-side technique to the posterior tibial artery and end-to-end anastomoses to the accompanying veins. The extremity was subsequently immobilized with a long-leg brace. Postoperatively, low-dose acetylsalicylic acid was administered for 72 h as antithrombotic prophylaxis, and no additional systemic anticoagulation was used.

**Figure 4:**
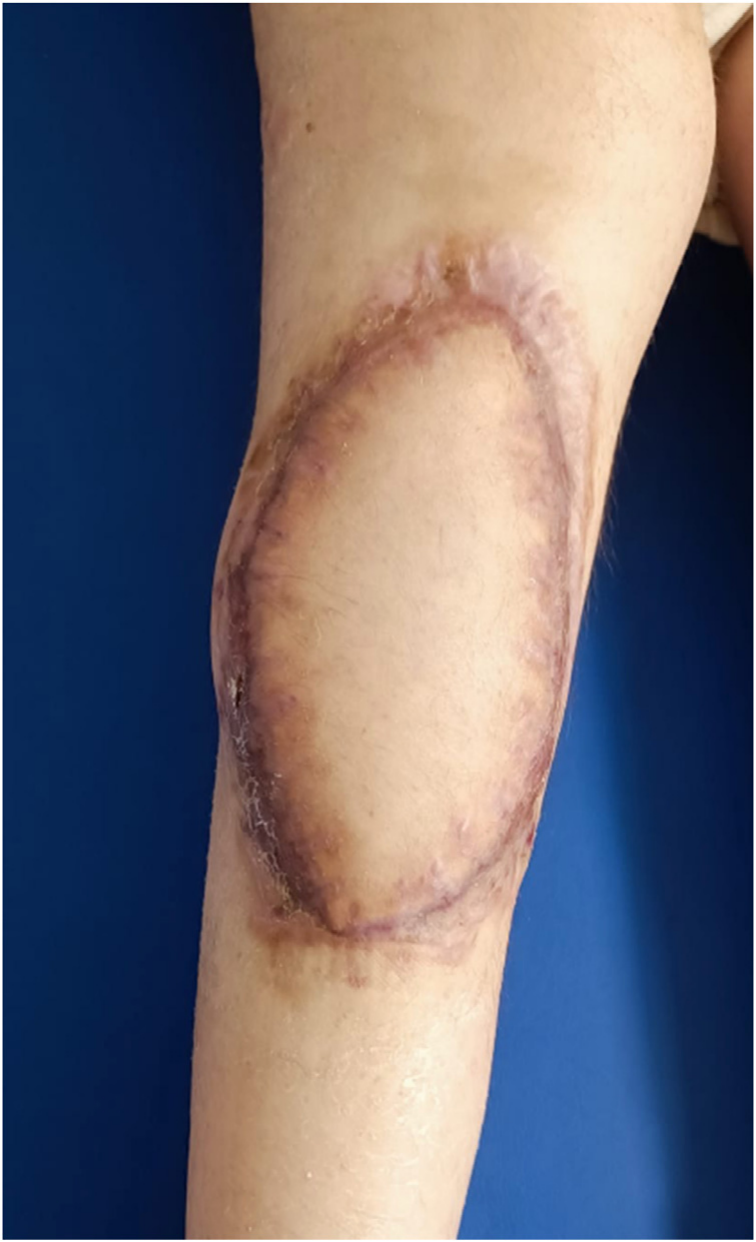
Representative view of the reconstruction at the 6-month follow-up.

No complications affecting flap viability were observed during either the early or late postoperative period. Although wound healing was delayed, complete and stable healing with full epithelialization and a satisfactory contour of the reconstructed area was achieved at the 6-month follow-up (Figure 4).

## Discussion

Spinal muscular atrophy (SMA) is characterized by progressive muscle atrophy secondary to lower motor neuron loss, resulting in marked reduction of muscle volume, fatty infiltration, and intramuscular fibrosis.^4^^,^^5^ These structural changes compromise soft tissue padding and regional perfusion, significantly reducing tissue tolerance to sustained external pressure. Consequently, patients with SMA are particularly susceptible to pressure-related skin breakdown, especially in the presence of limited mobility and immobilization devices.

Circular casts may further exacerbate this risk by increasing compartmental pressure and creating focal pressure peaks. In the setting of severe muscle atrophy, reduced limb–cast contact surface may lead to localized pressure concentration and rapid progression of pressure ulcers, underscoring the importance of vigilant monitoring and preventive measures in high-risk patients.^6^, ^7^, ^8^

Beyond mechanical factors, impaired muscle contraction, venous stasis, lymphatic dysfunction, and autonomic involvement may further compromise microcirculation in SMA, predisposing superficial lesions to rapid progression and delayed wound healing.^9^, ^10^, ^11^

Successful flap surgery depends on preserved anatomical planes and predictable perforator anatomy. In SMA, however, severe muscle atrophy and fibrosis distort fascial boundaries and intermuscular septa, rendering intramuscular perforator dissection unreliable and potentially unsafe.^12^, ^13^, ^14^

Microvascular free flap reconstruction has been reported in selected neuromuscular disorders such as post-polio syndrome and amyotrophic lateral sclerosis; however, these reports primarily emphasize perioperative management rather than disease-specific anatomical constraints.^15^^,^^16^ The lack of prior reports in SMA likely reflects the technical challenges imposed by profound muscle pathology. In the present case, these limitations necessitated a modified anterolateral thigh (ALT) flap harvest using antegrade pedicle dissection along the descending branch of the lateral femoral circumflex artery, with inclusion of a limited segment of vastus lateralis muscle to ensure pedicle safety.

In conclusion, patients with SMA are at increased risk for pressure ulcers following immobilization, and reconstructive planning must account for disease-specific anatomical alterations. With appropriate technical modifications and avoidance of intramuscular dissection at both donor and recipient sites, an ALT flap can be a safe and reliable reconstructive option in this challenging population.

## Funding

This study received no external funding.

## Ethical approval

Ethical approval was not required for this case report. Written informed consent for publication was obtained from the patient’s legal guardian.

## Declaration of competing interest

The authors declare no conflicts of interest.
